# Genome Sequence of an Extremely Drug-Resistant Clinical Isolate of *Acinetobacter baumannii* Strain AB030

**DOI:** 10.1128/genomeA.01035-14

**Published:** 2014-10-16

**Authors:** Peter C. Loewen, Yasser Alsaadi, Dinesh Fernando, Ayush Kumar

**Affiliations:** a Department of Microbiology, University of Manitoba, Winnipeg, Canada; b Department of Medical Microbiology, University of Manitoba, Winnipeg, Canada

## Abstract

We report the 4.3-Mbp genome sequence of a blood isolate of *Acinetobacter baumannii* strain AB030.

## GENOME ANNOUNCEMENT

*Acinetobacter baumannii* is notorious for causing infections that are very difficult to treat because of its high resistance to all classes of antibiotics (1, 2). Multidrug-resistant (MDR), extremely drug-resistant (XDR), and pandrug-resistant (PDR) isolates have been reported from around the globe (3–7). Here, we report the complete genome sequence of *A. baumannii* strain AB030, an XDR isolate from a bloodstream infection in a 29-year-old female (8). *A. baumannii* strain AB030 is resistant to aminoglycosides (amikacin, gentamicin); carbapenems (imipenem, meropenem); fluoroquinolones (ciprofloxacin, levofloxacin, moxifloxacin); penicillin and β-lactamase inhibitors (tazobactam-piperacillin); first-generation (cefzolin) and extended-spectrum (cefepime, ceftriaxone) cephalosporins; tigecycline; and trimethoprim-sulfamethoxazole (8). It is susceptible only to colistin (8). Therefore, we classified this isolate as XDR following the recommendations made by Magiorakos et al. (9).

DNA was isolated using the Ultra-Clean Microbial DNA isolation kit (MoBio Laboratories, Carlsbad, CA, USA) following the manufacturer’s instructions. Genome sequencing was performed using the PacBio platform at the Genome Quebec facility (Montreal, QC, Canada) using three SMRT cells. Assembly was carried out using the PacBio SMRT analysis pipeline version 2.2.0, with 93.3× coverage to give a single contiguous genome sequence. The sequence was annotated by the National Center for Biotechnology Information (NCBI) Prokaryotic Genomes Annotation Pipeline.

The genome consists of 4,335,793 bases with a G+C content of 39%. There are a total of 4,258 putative genes, which include 4,132 protein-, 18 rRNA-, and 73 tRNA-coding sequences.

### Nucleotide sequence accession number.

The genome sequence of *A. baumannii* AB030 was deposited in NCBI GenBank under the accession number CP009257.
